# [^99^Tc]Sestamibi bioaccumulation induces apoptosis in prostate cancer cells: an in vitro study

**DOI:** 10.1007/s11010-022-04439-8

**Published:** 2022-05-07

**Authors:** Nicoletta Urbano, Manuel Scimeca, Elena Bonanno, Rita Bonfiglio, Alessandro Mauriello, Orazio Schillaci

**Affiliations:** 1 Nuclear Medicine Unit, Department of Oncohaematology, Policlinico “Tor Vergata”, Viale Oxford 81, 00133 Rome, Italy; 2 Department of Experimental Medicine, Tor Vergata Oncoscience Research (TOR), University of Rome Tor Vergata, Via Montpellier 01, 00133 Rome, Italy; 3 San Raffaele Open University of Rome, Via di Val Cannuta 247, 00166 Rome, Italy; 4 Department of Experimental Medicine, University of Rome Tor Vergata, 00133 Rome, Italy; 5 Department of Biomedicine and Prevention, University of Rome “Tor Vergata”, Via Montpellier 01, 00133 Rome, Italy; 6 IRCCS Neuromed, Via Atinense, 18, 8607 Pozzilli, Italy

**Keywords:** Prostate cancer, Sestamibi, Apoptosis, Molecular imaging

## Abstract

The main aim of this preliminary in vitro study was to evaluate both the uptake of [^99^Tc]Sestamibi into prostate cancer cells and the relationship among [^99^Tc]Sestamibi bioaccumulation, cancer cells proliferation and apoptosis. An in vitro study in which PC3 prostate cancer cell line was cultured with increasing doses of decayed sestamibi has been developed. Specifically, PC3 cells were incubated with three different concentrations of [^99^Tc]Sestamibi: 10 µg/mL, 1 µg/mL, and 0.1 µg/mL Expression of apoptotic caspase-3 and AIF, as well as the ultrastructure of PC3 cells, were evaluated at T0 and after 24, 48, 72, and 120 h following [^99^Tc]Sestamibi incubation. Data here reported showed the bioaccumulation of sestamibi in prostate cancer cells. As concern the cancer cell homeostasis, the treatment of PC3 cells with [^99^Tc]Sestamibi strongly influenced the cells proliferation. Indeed, a significant reduction in the number of mitosis was observed. Noteworthy, the accumulation of sestamibi in prostate cancer cells was associated with the appearance of morphological signs of apoptosis. The increase in AIF and caspase 3 expression in prostate cancer cells treated with 10 µg/mL of [^99^Tc]Sestamibi confirmed that this radiopharmaceutical can trigger the apoptosis. To the best of our knowledge, this preliminary study reported for the first time in vitro data about the uptake of sestamibi in prostate cancer cells. The evidence about the accumulation of sestamibi in prostate cancer cells and its role in the apoptosis process could open new clinical perspectives on the use of this radiopharmaceutical in both the diagnosis and treatment of prostate cancers.

**Supplementary Information:**

The online version contains supplementary material available at 10.1007/s11010-022-04439-8.

## Introduction

Prostate cancer is one of the most frequent neoplasia that affects men in worldwide. In fact, the 2020 WHO data reported an incidence of prostate cancer of 14.1% in the male population with a prevalence of 20% [1]. In addition, prostate cancer represents the more frequent male neoplasia considering the > 60 years male population [1]. The onset of prostate cancer metastatic lesions often occurs several years after the first diagnosis. Thus, the development of new and ever more reliable diagnostic analysis capable to both predict the prostate cancer prognosis and early detect the occurrence of metastatic lesions is a great aim of the translational research.

Currently, the prognosis of prostate cancers is mainly based on the morphological evaluation of prostate biopsies and/or surgical samples [2]. Indeed, at the state of art only few molecular prognostic biomarkers have been identified for the prostate cancer [2]. Similarly, no/rare biological targets are available for precision therapy. In the last years, several researchers focused their attention of the evaluation of PSMA inhibitors as possible drugs capable to eradicate prostate cancer lesions also blocking cancer progression [3–5]. Some of these PSMA inhibitors are also radiolabeled and used to perform in vivo evaluation of prostate cancers in nuclear medicine departments [3–5]. The advantage of using radiolabeled molecules is the possibility to manage the patients during the post-diagnosis period without the need for biopsy sampling [6–8] and even to develop theragnostic approaches in which the same molecules can be used for both diagnosis and therapy by changing its concentrations or the linked radionuclide [9]. However, many of these molecules are in clinical trials still.

Hence, the identification of radiolabeled molecules showing a significant uptake in prostate cancer lesions could improve the currently armamentarium available to clinicians for managing prostate cancer patients.

A possible investigation strategy is to evaluate the specificity and sensibility for prostate cancer lesions of radiolabeled molecules already used for diagnosis or therapy of other cancers such as breast neoplasia. This strategical approach is based on the knowledge of the molecular mechanisms that drive-specific radiolabeled molecules into cancer cells.

In this context, [^99^Tc]Sestamibi, a radiolabeled molecule used for detecting breast cancer lesions by molecular scintigraphy, could in vivo mark also prostate cancer lesions. It is known that sestamibi uptake mainly occurs to passive diffusion through biological membranes with possible accumulation into the mitochondria since the positive charge of the lipophilic structure of sestamibi [10, 11]. In our recent study, we demonstrated that sestamibi uptake is greater in breast cancer lesions with high propensity to form bone metastasis due to the presence of osteoblast-like cells rich in mitochondria [12, 13]. Similarly, we also demonstrated that the development of bone metastasis from prostate cancer is related to the presence of prostate cancer lesions characterized by the presence of osteoblast like cells [14–16]. Thus, it is possible to hypothesize a similar uptake of [^99^Tc]Sestamibi in prostatic lesions. In addition, recent evidences suggest that sestamibi bioaccumulation can induce the apoptosis of cancer cells by altering the mitochondrial structures. This allows to speculate about its use as theragnostic agent [17]. In fact, the capability of sestamibi to trigger the apoptotic process seem to be related to the concentration of molecules accumulated in the cells [17].

Starting from these considerations, the main aim of this in vitro study was to evaluate both the uptake of [^99^Tc]Sestamibi into prostate cancer cells (PC3) and the relationship among [^99^Tc]Sestamibi bioaccumulation, cancer cells proliferation and apoptosis. To this end, an in vitro study in which PC3 prostate cancer cell line and BT-474 breast cancer cells (surrogate control) were cultured with increasing doses of decayed sestamibi has been developed. At the end of experimental phases, ultrastructural, microanalytical and immunocytochemical analysis were performed.

## Materials and methods

All methodologies and experimental procedures here described were achieved in agreement with the last Helsinki Declaration.

### Cell Culture

PC3 and BT-474 cells was obtained from the American Type Culture Collection (ATCC. Manassas, Virginia, USA) and maintained by the Cell and Tissue Culture Core, Lombardi Cancer Center (Reservoir Rd. NW Washington D.C. 20057, USA). Cells were routinely cultured in DMEM high glucose (Sigma-Aldrich, St. Louis, Missouri, USA) supplemented with 10% fetal bovine serum (FBS).

In detail, cells from the first or second passage were seeded into a 24-well plate at a density of 30 × 10^3^ cells/well. Then, both PC3 and BT-474 cells were incubated with: (a) [^99^Tc]Sestamibi 10 µg/ml (b) [^99^Tc]Sestamibi 1 µg/ml and (c) [^99^Tc]Sestamibi 0,1 µg/ml. The expression of both Ki67 and caspase 3 were evaluated at T0 and after 24, 48, 72 and 120 h after sestamibi incubation. Cells treated with the vehicle were used as control (CTRL).

Cell proliferation was investigated by counting the number of mitotic cells for each time point at the time 0 and after 72 h. Morphology was studied both toluidine blue staining.

### Immunocytochemistry

Immunocytochemistry was performed to investigate the expression of caspase 3 and Apoptosis-Inducing Factor (AIF) on both PC3 and BT-474 cells treated with [^99^Tc]Sestamibi. Both reactions were evaluated by immunofluorescence staining.

Cancer cells were plated on poly-l-lysine-coated slides (Sigma-Aldrich cat #P4707) in 24-well cell culture plates and fixed in 4% paraformaldehyde. After pre-treatment with EDTA citrate at 95 °C for 20 min and 0.1% Triton X-100 for 15 min, cells were incubated 1 h with the anti-AIF rabbit monoclonal antibody (clone E-20, Abcam, Cambridge, UK) and anti-caspase 3 mouse monoclonal antibody (31A1067, Novus Biologicals, USA). Washings were performed with PBS/Tween20 pH 7.6. Reactions were revealed by using an TexasRed conjugate anti-rabbit antibody for AIF and an TexasRed conjugate anti-rabbit antibody for the caspase 3.

Reactions were evaluated by counting the number of AIF or caspase 3-positive cells on 100 in total in randomly selected regions.

### TEM and EDX analysis of cell cultures

Cells were fixed in 4% paraformaldehyde, post-fixed in 2% osmium tetroxide and embedded in Epon resin for morphological studies. After washing with 0.1 M phosphate buffer, the sample was dehydrated by a series of incubations in 30%, 50%, and 70%, ethanol. Dehydration was continued by incubation steps in 95% ethanol, absolute ethanol, and hydroxypropyl methacrylate, then samples were embedded in Epon (Agar Scientific, Stansted Essex, UK).

Eighty micrometre ultra-thin sections were mounted on copper grids and observed with Morgagni FEI 268D transmission electron microscope (FEI Company) to study the mitochondria ultrastructure.

Unstained ultra-thin sections of approximately 100-nm-thick were mounted on copper grids for microanalysis. EDX spectra were acquired by using an EDX detector (Thermo Scientific, Waltham, MA, USA) [18, 19].

### Statistical analysis

Statistical analysis was performed using GraphPad Prism 5 Software (San Diego, CA, USA). The number of mitosis and immunohistochemical data were analyzed by the Kruskal–Wallis test (*p* < 0.05) and by Mann–Whitney test (*p* < 0.0005).

## Results

### Effect of sestamibi accumulation on cancer cells proliferation

**Fig. 1:**
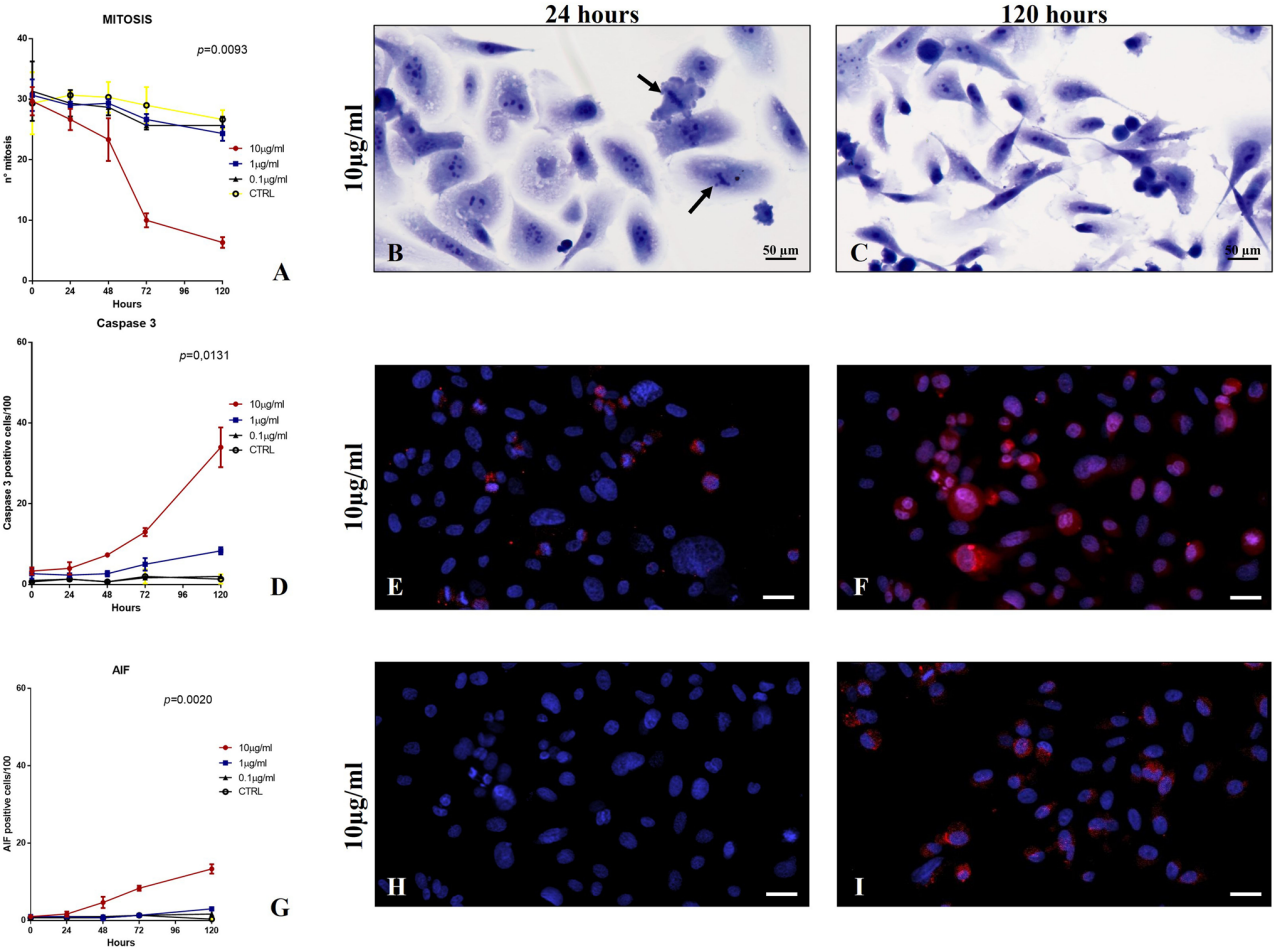
Evaluation of Mitosis and apoptotic phenomenon in PC3 cell lines. **A** The graph shows the number of mitosis in PC3 cancer cells after sestamibi treatment. **B** Image displays two mitoses (arrows) in PC3 cells treated for 24 h with sestamibi. **C** After 120 h of sestamibi treatment no/rare mitosis are observed. **D** The graph shows the number of caspase 3 positive cells after sestamibi treatment. **E** Few caspase 3 positive PC3 cells after 24 h of sestamibi treatment. **F** Numerous caspase 3 positive PC3 cancer cells after 120 h of sestamibi treatment. **G** The graph shows the number of AIF-positive cells after sestamibi treatment. **H** After 24 h of the treatment with sestamibi no/rare AIF positive cells are detected. **I** Several caspase 3 positive PC3 cancer cells after 120 h of sestamibi treatment. Scala bar represents 100 µm

As concern the cell proliferation, a significant decrease in the number of mitosis was observed in cell cultures incubated with both 10 µg/ml and 1 µg/ml of [^99^Tc]Sestamibi if compared with cell treated with a concentration of 0.1 µg/ml (Fig. 1A–C). Indeed, data here reported showed that low concentration of [^99^Tc]Sestamibi (0.1 µg/ml) does not influence the cell proliferation (Fig. 1A–C). Cell cultures treated with 0.1 µg/ml [^99^Tc]Sestamibi displayed a level of cell proliferation like that observed in the controls (Fig. 1A). This datum can explain the association observed between the sestamibi uptake and the percentage of mitotic cancer cells in vivo. It is important to note that high concentration of 99mTC-sestamibi (10 µg/ml) induced a significant reduction of the proliferation index already after 24 h of treatment (Fig. 1A).

### Effect of sestamibi accumulation on cancer cells apoptosis

One-way ANOVA showed significant data distribution for both the number of caspase 3 (*p* = 0.0131) and AIF (*p* = 0.0020) positive cells in the experimental groups (Fig. 1D–I). Noteworthy, high concentration of [^99^Tc]Sestamibi (10 µg/ml) induced a significant increase in the number of apoptotic cells (caspase 3 or AIF-positive cells) if compared with all others experimental conditions, included the controls (Fig. 1D, G). Specifically, a great increase in the number of caspase 3 positive cells was observed after 48 h (Fig. 1A, D). This datum suggests that only at high concentration the sestamibi could be able to induce the apoptotic process by regulation the caspase 3 signal.

### Proliferation vs apoptosis

**Fig. 2:**
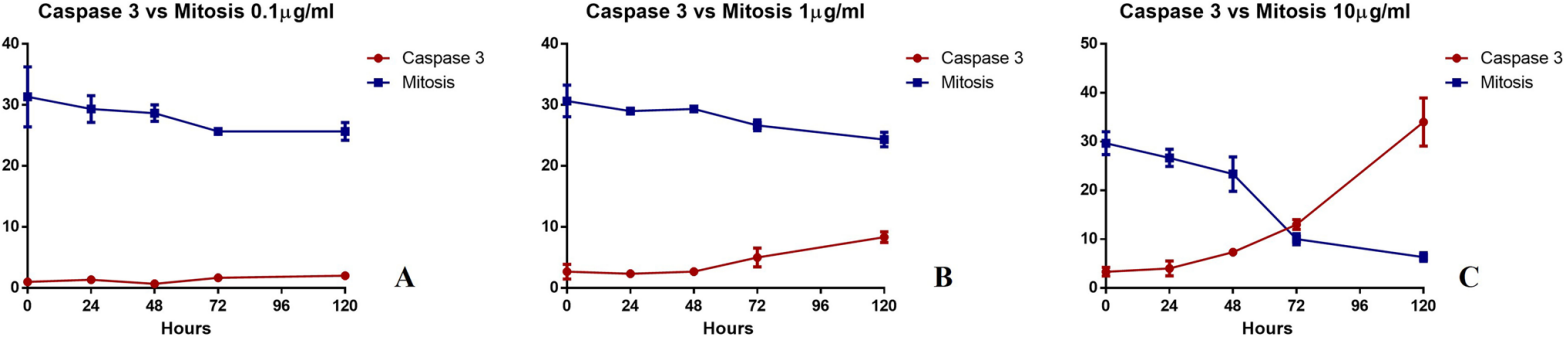
Comparison between caspase 3 expression and mitosis in PC3 cancer cells treated with [^99^Tc]Sestamibi. **A** Graph shows the number of both caspase-3 positive cells and mitosis in PC3 cells after 24, 48, 72, 96, and 120 h of [^99^Tc]Sestamibi incubation (10 µg/mL). **B** Graph displays the number of both caspase-3 positive cells and mitosis in PC3 cells after 24, 48, 72, 96, and 120 h of [^99^Tc]Sestamibi incubation (10 µg/mL). **C** Graph shows the number of both caspase-3 positive cells and mitosis in PC3 cells after 24, 48, 72, 96, and 120 h of [^99^Tc]Sestamibi incubation (10 µg/mL)

To investigate the effect of sestamibi on prostate cancer cell proliferation and apoptosis a comparison between caspase 3 expression data and the number of mitotic cells was performed (Fig. 2).

Specifically, it was clear that only using the 10 µg/ml of [^99^Tc]Sestamibi concentration a complete reversion between proliferation (number of mitosis) and apoptosis (caspase 3 expression) was obtained (Fig. 2A–C). In particular, after 72 h the number of caspase 3 positive cells were higher than those mitotic cells thus suggesting an imbalance capable to arrest the tumour proliferation (Fig. 2C). Similar data was observed in the BT474 breast cancer cell lines (see supplementary).

### Transmission electron microscopy and microanalysis

**Fig. 3:**
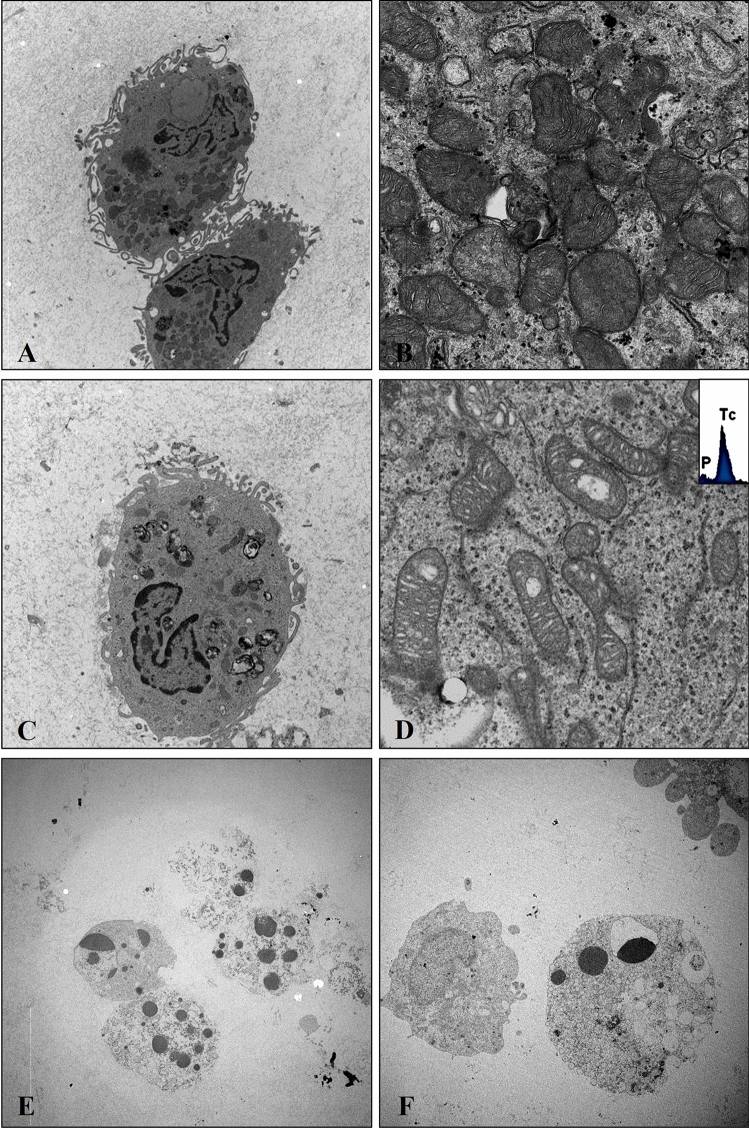
Transmission electron microscopy and microanalysis. **A** PC3 Prostate cancer cells rich in mitochondria after 48 h of sestamibi treatment. Scale bar represents 20 µm. **B** High magnification of panel A displays no mitochondrial alterations. Scale bar represents 2 µm. **C** PC3 Prostate cancer cells rich after 72 h of sestamibi treatment. Scale bar represents 20 µm. **D** High magnification of panel C displays moderate mitochondrial alterations. EDX spectrum demonstrated the presence of Technetium (Tc) into the degenerated mitochondria. Scale bar represents 2 µm. **E**, **F** Several apoptotic PC cancer cells after 120 h of sestamibi treatment. Scale bar represents 20 µm

Transmission electron microscope analysis of PC3 cell cultures treated with10µg/ml showed no significant ultrastructural alteration after 24 h (Fig. 3A, B). After 72 h of treatment cells frequently displayed alterations in the mitochondrial ultrastructure (Fig. 3C, D). Moreover, these mitochondria were often characterized by the presence of Technetium (Tc) (Fig. 3D). Numerous apoptotic cells were observed after 120 h of treatment instead (Fig. 3E–F).

## Discussion

Although preliminary, data here reported demonstrated that in vitro sestamibi bioaccumulation induces apoptosis in a prostate cancer cell line. Indeed, the treatment of PC3 prostate cancer cells with increasing concentration of decayed [^99^Tc]Sestamibi was able to both reduce cell proliferation and trigger the apoptosis.

The identification of new molecules capable to detect prostate cancer lesions represents one of the most important topics of the scientific research. The management of prostate cancer patients requires the continuous development of clinical investigations capable to follow the patients for several years after the diagnosis and/or treat the lesions with high metastatic potential. In fact, the onset of prostate cancer metastatic lesions often occurs several years after the first diagnosis. Currently, the follow-up for prostate cancer patients includes the use of serological tests [20], such as the blood evaluation of Prostate Specific Antigen (PSA) [20], or imaging analysis by Fluorodeoxyglucose (FDG)-Positron Emission Tomography (PET) [21] or 18 F-choline PET/Computer Tomography [22]. The PSA blood evaluation is a non-invasive test, but its values are influenced by numerous variables such as systemic inflammation and prostatitis [23]. On the contrary, molecular imaging analysis is very sensitive but more invasive, respect to blood tests.

Thus, the identification of less invasive diagnostic methods with low radiation risks, if compared with [^18^F], could improve the current armamentarium available to physicians in the fight against the prostate cancer.

In this context, the low radiation exposure related to Single Photon Emission Computed Tomography (SPECT) investigations [24] might allow to design a follow-up protocol in which gamma-emitted radiolabeled molecules can be used to early detect both primary lesions and metastatic ones. In addition, the same molecules could be used for theragnostic protocols by changing their concentration and/or the related radionuclide [25].

According to this, several gamma-emitted molecules have been tested as possible radiotracer for SPECT analysis [26]. Our recent investigations highlighted the fundamental role of [^99^Tc]Sestamibi, in the detection of breast cancer lesions with high propensity to form bone metastasis [12, 13]. Moreover, in an in vitro study the bioaccumulation of [^99^Tc]Sestamibi was able to induce the cancer cell apoptosis in a breast cancer cell line [17].

Starting from these evidences, and even considering the similarity of breast and prostate cancer in terms of morphology, origin and progression, especially for the development of bone metastasis, the main aim of this in vitro study was to evaluate both the uptake of [^99^Tc]Sestamibi into PC3 prostate cancer cells and the relationship among [^99^Tc]Sestamibi bioaccumulation, cancer cells proliferation and apoptosis. To this end, an in vitro study in which PC3 prostate cancer cell line and BT-474 breast cancer cells (surrogative ctrl) were cultured with increasing doses of decayed sestamibi has been developed.

Noteworthy, data here reported, although preliminary, demonstrated the bioaccumulation of sestamibi in prostate cancer cells. Specifically, transmission electron microscopy and EDX microanalysis investigations showed the presence of technetium in both the cytoplasm and mitochondria of cancer cells.

As concern the cancer cell homeostasis, the treatment of PC3 cells with [^99^Tc]Sestamibi strongly influenced the cells proliferation. Indeed, a significant reduction in the mitosis was observed. Remarkable, the decrease of cell proliferation was inversely proportional to the concentration of [^99^Tc]Sestamibi.

In line with these data, the accumulation of sestamibi in prostate cancer cells was associated with the appearance of morphological signs of apoptosis. The immunocytochemical analysis of AIF, a mitochondrial oxidoreductase capable to trigger the cell death process [27], and caspase 3, a caspase protein that interacts with caspase-8 and caspase-9 sustaining the canonical intrinsic mitochondrial death pathway [28], confirmed that the treatment with [^99^Tc]Sestamibi can induce the canonical apoptosis.

The concomitant increase in the expression of caspase3 and AIF suggests the mechanism in which [^99^Tc]Sestamibi may induce the apoptosis. In fact, it has been demonstrated that the caspase3 can induce the permeabilization of the outer mitochondrial membrane, thereby triggering the release of AIF [29]. Hence, the [^99^Tc]Sestamibi bioaccumulation can induce an increase of caspase3 expression related to a severe mitochondrial damage which in turn is responsible for the release of AIF. This hypothesis is supported by the mitochondrial alteration observed during the ultrastructure investigation in cancer cells treated with high concentration of [^99^Tc]Sestamibi. Another possible mechanism could involve the increase in Reactive oxygen species (ROS) as a result of the mitochondrial damage caused by [^99^Tc]Sestamibi accumulation. Indeed, it is well known that the ROS may trigger the molecular events related to the apoptosis [30].

As described in our previously investigations, [^99^Tc]Sestamibi induced an increase of apoptotic cells in a breast cancer cells line.

## Limits of the study

This is the first study concerning the effect of [99Tc]Sestamibi bioaccumulation on prostate cancer cells. Despite the preliminary nature of the investigation the results here showed coul have a great impact on the identification of new radiolabeled drugs for the management of prostate cancer patients.

It has not escape to our notice that further investigations are needed to confirm data here reported. In particular, the effects of [99Tc]Sestamibi accumulation on prostate cancer cells should be further investigated using in vivo animal models.

## Conclusion

To the best of our knowledge, this study for the first time reported in vitro data about the uptake of sestamibi in prostate cancer cells. The evidence about the accumulation of sestamibi in prostate cancer cells and its role in the apoptosis process can open new clinical perspectives on the use of this radiopharmaceutical in both the diagnosis and treatment of prostate cancers. If confirmed by further ex vivo and in vitro studies, the capability of sestamibi to induce apoptosis of prostate cancer cells can lay the scientific rationale for considering this molecule as a theragnostic agent. Lastly, these investigations further highlight the fundamental cooperation between nuclear medicine, pathology and cellular biology in both research and clinical studies [31–33].

## Supplementary Information

Below is the link to the electronic supplementary material.

Evaluation of Mitosis and apoptotic phenomenon in BT474 cell lines. A) The graph shows the number of mitosis in BT474 cancer cells after sestamibi treatment. B) The graph shows the number of caspase 3 positive cells after sestamibi treatment. C) The graph shows the number of AIF positive cells after sestamibi treatment. Supplementary file1 (JPG 546 kb)

## Data Availability

All data were included in the manuscript.
