# Supplementary figures and images for: [99Tc]Sestamibi bioaccumulation induces apoptosis in prostate cancer cells: an in vitro study
Source: Mol Cell Biochem. 2022 May 7;477(10):2319–26. doi: 10.1007/s11010-022-04439-8 (PMC9499905; doi:10.1007/s11010-022-04439-8)

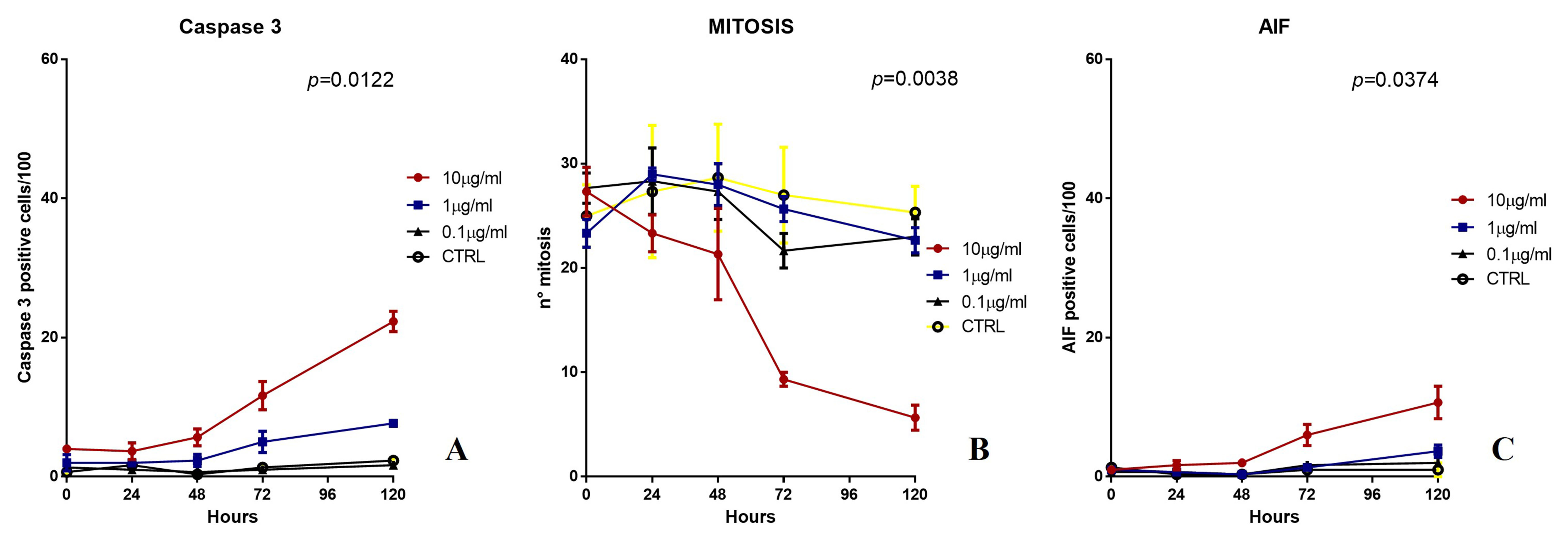

Supplement: Supplementary file 1 — Evaluation of Mitosis and apoptotic phenomenon in BT474 cell lines. A) The graph shows the number of mitosis in BT474 cancer cells after sestamibi treatment. B) The graph shows the number of caspase 3 positive cells after sestamibi treatment. C) The graph shows the number of AIF positive cells after sestamibi treatment. Supplementary file1 (JPG 546 kb) [file 11010_2022_4439_MOESM1_ESM.jpg]
